# Widespread Availability of Artemisinin Monotherapy in the United States

**DOI:** 10.3201/eid1705.101532

**Published:** 2011-05

**Authors:** Robert M. Rakita, Uma Malhotra

**Affiliations:** Author affiliations: University of Washington, Seattle, Washington, USA (R.M. Rakita);; Virginia Mason Medical Center, Seattle (U. Malhotra)

**Keywords:** malaria, Plasmodium falciparum, artemisinins, drug resistance, microbial, letter

**To the Editor**: Artemisinin-based combination therapies are recommended as first line treatments for *Plasmodium falciparum* malaria in most areas of the world. The article by Shahinas et al. (*1*) describes a patient who had *P. falciparum* malaria after returning from Nigeria. Her isolate had an elevated 50% inhibitory concentration to artemisinin derivatives. She had obtained artesunate in Nigeria and took it weekly for malaria prophylaxis, which might have contributed to the relative resistance found.

In 2009, one artemisinin-based combination therapy (artemether/lumefantrine) became available for use in the United States. However, it is not widely appreciated that artemisinin is actually available in the United States as an herbal supplement for over-the-counter purchase (*2*). It is marketed for general health maintenance and for treatment of parasitic infections and cancers (Figure), although as with other supplements it is not intended to diagnose, treat, cure, or prevent any disease. As in the patient described by Shahinas et al., widespread use of artemisinin or its derivatives as monotherapies could potentially lead to progressively increasing resistance in *P. falciparum* malaria (*3*). Studies in western Cambodia, where artemisinin monotherapy has been available for many years, have revealed in vivo artesunate resistance, with markedly decreased parasite clearance times (*3*). Progressive spread of artemisinin resistance could have disastrous consequences for the global control of malaria. Thus, minimally regulated use of potent compounds in dietary supplements has the potential for major public health implications.

**Figure:**
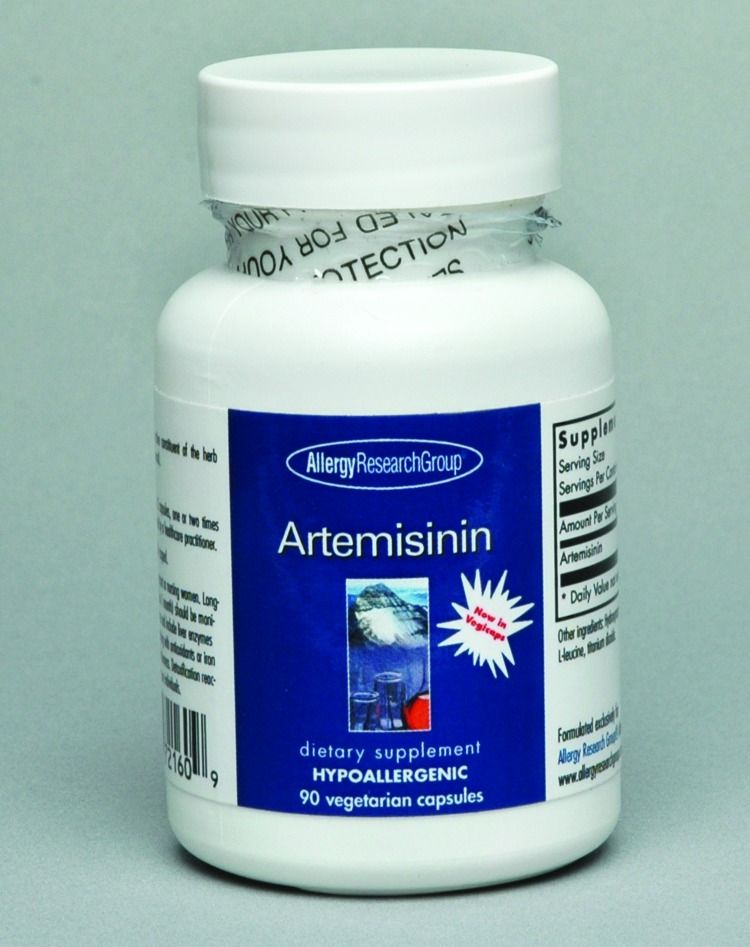
Bottle of artemisinin, available over-the-counter as an herbal supplement.
